# Clothing

**Published:** 1877-10

**Authors:** 


### CLOTHING.

We have come to believe that cases of serious sickness are not infrequent where the first cause of the malady can be directly traced to imperfect clothing. Now that the cold weather is approaching, it becomes us to consider this matter, to the end that we may not only accomplish it cheaply and fashionably, but more sensibly—healthfully.

Let us commence with the head:—What sort of a hat should a school boy or girl wear? The *best*, for either sex, should be made of wool—crocheted with soft, fleecy ear-rosettes, to keep the ears warm, when the freezing weather calls for such protection. Never cover a child's head with beaver, felt, or other impervious material, or, for that matter, a grown person's either, for a circulation of air must be had through the hat or cap in order that the perspiration can be carried off, and the breathing apparatus of the skin not be interfered with, if you would avoid headaches and bald scalps. Be sure that the hat or cap be supplied with some sort of a fore-piece to shield the eyes from the sun, otherwise will the eyes suffer from the glare of the strong sunlight striking directly upon them.

Next, keep your hands, feet and belly warm. Woolen gloves and stockings, the latter changed daily, and a broad woolen band over the bowels, will protect you from likelihood of sickness from a freezing temperature. Be careful to have your stockings fit well. Do not have them come to a point at the toes, but have them give the toes plenty of room, that they may not be forced to override one another and thus eventually incur deformity. Let the shoes be broad on the soles, flexible, and not snug about the ankles—tightness about the ankles renders those useful joints weak and liable to turn, subjecting you to the liability of a few months gymnastic exploits upon crutches.

Do not fasten the stockings of your child to the legs with elastics. They arrest the circulation and prevent the natural warming from the free circulation of the blood. Fasten them by means of buttons and loops attached to bands reaching to the waist-band.

If the child be slender or delicate, or has a languid circulation, have him wear woolen, instead of cotton, shirts, and place also an extra thickness of flannel over the chest. The flannel exerts a gentle friction upon the skin, increases the cutaneous circulation, and so warms the body. It also keeps an even temperature of the body, rendering less likely any disturbance from sudden changes of weather.

Never permit your child to brave the winter weather with limbs incased in cotton hose, and shoulders covered only with a cotton dress, and that too short at both ends; with legs bare above the tops of the stockings, until some thin fabric is reached a few inches above the stocking tops; but dress them warmly, in woolen, with skirts falling below the knees, and body cut high in the neck, and thick woolen drawers underneath, then stick on all the feathers, furbelows and gimcracks over all, your fancy may suggest.

In the next room, our two daughters are having considerable additions made to their winter's wardrobe. But, when they attend their first evening party, be assured they will *not* appear in short-sleeved and low-necked dresses, unless a reasonable thickness of flannel first covers their arms and bodies. No impropriety of dress has been more provocative of sickness, and often, too, to fatality, than the abominable party dresses referred to. Parents are doing their lovely daughters an injustice and inflicting much trouble upon themselves, by permitting such a violation of the rules of health, if not of common decency, as is illustrated in the sort of garments named.

Perfect health, during the winter season, depends much upon the selection of our clothing. Let it be *warm*, even at the sacrifice of every other consideration.

A French author says:—"When I lost my wife, every family in the town offered me another; but when I lost my horse no one offered to make him good."

When Sir Walter Scott was urged not to prop the fallen credit of one of his acquaintances, he replied:—"The man was my friend, when my friends were few, and I will be his now that his enemies are many."

